# Proteomic Analysis of TCGA Data Reveals Limited Prognostic Value of p53 but Suggests BAX as a Potential Survival Marker in Cervical Carcinoma

**DOI:** 10.3390/ijms27156717

**Published:** 2026-07-27

**Authors:** Sebastian M. Toennießen-Klein, Dennis Rangno, Abhirami Sunil, Maria Bozko, Przemyslaw Bozko

**Affiliations:** 1 Department of Internal Medicine I, University Hospital Tübingen, 72076 Tübingen, Germany; 2 The M3 Research Center, Eberhard-Karls Universität Tübingen, 72076 Tübingen, Germany; 3 Institute of Genetics and Biotechnology, Faculty of Biology, University of Warsaw, 02-106 Warsaw, Poland

**Keywords:** cervical cancer, HPV, p53, patient survival, diagnostic importance, Cancer Genome Atlas

## Abstract

Cervical carcinoma is predominantly driven by high-risk human papillomavirus (HPV) infection, in which functional inactivation of p53 occurs mainly through viral oncoproteins rather than mutations in the *TP53* gene. This raises questions concerning the prognostic relevance of *TP53* gene status and p53 protein levels in this tumor type. This study aimed to systematically evaluate the prognostic significance of *TP53* mutation status, p53 protein abundance, and selected downstream p53-regulated proteins in cervical carcinoma, with particular focus on tumors carrying wild-type *TP53*. Clinical and proteomic data from 162 cervical carcinoma patients were retrieved from The Cancer Genome Atlas (TCGA). Protein levels (p53, BAX, p21/CDKN1A, and TIGAR) were assessed using reverse-phase protein array (RPPA) data. Survival analyses were performed using Kaplan–Meier estimates with log-rank testing after stratification into quartile-based expression groups. The vast majority of tumors (~93%) harbored wild-type *TP53*, and the *TP53* mutation status showed no association with patient survival. In tumors with wild-type *TP53*, p53 protein levels did not significantly correlate with overall survival, indicating a limited prognostic value. In contrast, elevated levels of the pro-apoptotic protein BAX showed a clear tendency to be associated with poorer overall and progression-free survival, suggesting BAX as a potential prognostic marker in this specific molecular context. The expression of p21/*CDKN1A* showed no prognostic relevance, while high TIGAR levels displayed a slight trend toward poorer survival, although without reaching statistical significance. In summary, in HPV-driven cervical carcinoma, neither the *TP53* mutation status nor the p53 protein abundance reliably predict patient outcome. Instead, selected downstream effectors of p53 signaling—particularly BAX—may provide more informative prognostic insights in tumors retaining wild-type *TP53*. These findings highlight the importance of assessing functional outputs of p53 signaling rather than the p53 status alone in this disease context.

## 1. Introduction

The p53 protein was discovered more than four decades ago simultaneously in several laboratories [1,2,3]. Since then, p53 has become one of the most extensively studied proteins in biology. The reason for this prominent role is its involvement in nearly all types of cellular stress, where it coordinates cellular responses to these stimuli [4,5,6,7]. Today, it is well established that p53 possesses multiple biochemical functions, making it one of the major barriers against cancer development, both at the stages of tumor initiation and progression, as well as in the response to anticancer treatment. From a biochemical perspective, the primary function of p53 is that of a sequence-specific transcription factor (TF). The p53 protein binds to specific DNA sequences within the genome (p53 response elements), leading to the activation of transcription of target genes [8]. In addition, p53 can also repress the transcription of various genes, typically through indirect mechanisms [9]. In addition to its tumor-suppressing functions, p53 has been implicated in many other aspects of mammalian physiology [4]. For example, p53 plays an important role in cellular energy metabolism [10,11,12] as well as in the regulation of host–microbiome interactions [13,14] and the control of ferroptosis [15,16]. To fulfill these diverse functions, p53 is not limited to its role as a transcription factor. Indeed, an increasing body of evidence indicates that p53 also exerts transcription-independent activities [17,18,19]. Many of these functions seem to be mediated not by nuclear but by cytoplasmic p53, even though actual preservation of activity of the cytoplasmic state remains controversially discussed [19,20,21].

One of the most important functions that has been reported to be regulated by cytoplasmic p53 is the control of apoptosis through its association with mitochondria. Permeabilization of the mitochondrial membrane in response to various stimuli is regulated by interactions between pro- and anti-apoptotic factors associated with this organelle, for example p53, which has been shown to localize to the mitochondria under numerous cell death-inducing conditions, as reviewed by Kroemer et al. [22]. Another important example of cytoplasmic p53 function is the regulation of autophagy. Autophagy can be defined as the sequestration and degradation of portions of the cytoplasm, enabling the removal of damaged cellular components. Although autophagy can, in some contexts, lead to cell death, it more commonly serves as an adaptive mechanism that allows cells to survive under stress conditions rather than undergoing lethal self-digestion [23]. By removing damaged organelles, which may serve as sources of reactive oxygen species, autophagy plays an important role in maintaining genomic integrity [24]. Although p53 can transcriptionally activate several genes that induce autophagy, such as *DRAM* [25] and SESTRIN-1 and -2 [26], in most cases the suppression of autophagy is mediated by cytoplasmic rather than nuclear p53 [27].

Taken together, these observations indicate that p53 should be regarded not only as the classical “guardian of the genome” but also as a “guardian of the cell.” Given its central role in numerous cellular processes, p53 activity must be tightly regulated. Indeed, p53 is controlled by a wide range of proteins. These regulatory mechanisms primarily involve post-translational modifications such as ubiquitination and phosphorylation [28,29]. Considering the involvement of p53 in numerous biochemical pathways, several studies have compiled comprehensive lists of genes regulated by p53, as systematically reviewed in [30]. In that work, the authors established a hierarchical list of positively regulated target genes that are important for both cell cycle control and cell death or survival. In the present study, we focused on the top 20 positively regulated genes, given that one of the primary functions of p53 is to control the transcription of genes involved in cell cycle regulation and apoptosis. Traditionally, most studies have assessed the expression of p53 target genes at the mRNA level. However, we consider it more informative to analyze protein levels, as proteins—not mRNA—are the direct effectors of cellular functions involved in tumor progression and cellular responses to stress, including anticancer therapy. Accordingly, we examined the protein expression levels of the top 20 p53-regulated genes. Complete datasets linking protein expression with patient survival were available for three of these candidates. Among them, BAX is one of the most prominent p53 target genes. It encodes a pro-apoptotic protein that plays a key role in the mitochondrial pathway of apoptosis [31]. The p21 protein (encoded by the gene *CDKN1A*) plays an important role in maintaining genomic stability by functioning as a cyclin-dependent kinase inhibitor, thereby inducing cell cycle arrest in the G1 phase in response to DNA damage or the absence of growth factors [32,33]. Finally, the *TP53*-induced glycolysis and apoptosis regulator (TIGAR) modulates glycolysis and promotes DNA damage repair and cell proliferation. The biochemical functions of TIGAR include the regulation of oxidative stress, reduction in inflammation, modulation of mitochondrial function, and inhibition of apoptosis [34].

Cervical carcinoma is a highly aggressive malignancy that develops through a series of preneoplastic lesions collectively defined as cervical intraepithelial neoplasia (CIN) grades I, II, and III [35]. It is well established that high-risk human papillomavirus (HR-HPV) types are strongly associated with invasive squamous cell carcinoma (ISCC) of the cervix [35]. Among these, the HPV types 16 and 18 are the most frequently detected in such lesions [36,37]. Integration of HR-HPV DNA into the host cell genome leads to the expression of the viral oncoproteins E6 and E7. These proteins interfere with cell cycle regulation by targeting key tumor suppressors, with E6 promoting the degradation of p53 and E7 inactivating the retinoblastoma protein (pRb) [36]. This disruption results in loss of cell cycle control, chromosomal instability, and ultimately cervical carcinogenesis [38]. Moreover, detection of HPV infection has become one of the most important biomarkers in cervical cancer screening and management [39,40,41,42,43,44,45,46,47,48]. The inactivation of p53 occurs through the formation of a complex between p53 and an E6-associated protein (E6AP), an E3 ubiquitin ligase recruited by the E6 oncoprotein. This interaction leads to ubiquitination-mediated degradation of p53, a mechanism that has been extensively documented in cervical carcinoma and other HPV-associated malignancies [49,50,51,52,53,54,55,56,57,58]. Considering the central role of p53 in regulating cell proliferation, cell death, and responses to various forms of cellular stress [4,5,6], numerous researchers have attempted to use *TP53* mutation status and p53 protein levels as prognostic markers in cervical carcinoma. However, the results of these studies have been inconsistent, largely due to methodological differences and variability in analytical approaches. In addition, many of these studies were conducted on relatively small patient cohorts, limiting the robustness of their conclusions. The only widely accepted observation is that *TP53* mutations are relatively rare in cervical cancer [59,60]. In contrast, findings regarding the prognostic significance of p53 protein levels remain highly inconsistent. Many researchers and clinicians have investigated potential correlations between *TP53* polymorphisms [61,62,63], p53 protein expression, and patient survival [51,56,58,64,65,66,67,68,69,70,71,72,73,74,75,76,77,78,79,80,81,82]. To provide more clarity, we have categorized previously reported findings on the relationship between p53 protein levels and clinical outcomes in cervical carcinoma into distinct groups.

### 1.1. High p53—Poor Prognosis

Many researchers and clinicians have reported that elevated p53 levels in tumor tissues are significantly associated with a poor prognosis. A notable meta-analysis performed by Zhou and colleagues, based on 11 published studies including 1105 patients, demonstrated that p53 overexpression has an unfavorable impact on overall survival [64]. Similar findings were reported by Lu and colleagues, who analyzed 228 cases of cervical cancer. In their study, p53 expression was assessed by immunohistochemistry using tissue microarrays. Statistical analysis revealed that the positive expression rate of p53 in cervical cancer was significantly higher than in CIN and cervicitis groups. Furthermore, p53 expression correlated with histological type and tumor grading. Importantly, elevated p53 protein levels in tumor tissues were associated with reduced patient survival [77]. Consistent results were obtained by Huang and colleagues, who showed that overexpression of p53 in cervical carcinoma samples predicts a poor clinical outcome [67]. Specifically, p53 nuclear staining was absent in normal epithelium and low-grade squamous intraepithelial lesions (LSILs), whereas most invasive carcinomas were positive. The mean p53 expression level was significantly higher in invasive carcinoma compared with normal tissue, precancerous lesions, and microinvasive carcinoma. Moreover, p53 overexpression was significantly associated with advanced tumor stage, leading the authors to propose p53 as a predictor of poor prognosis [67]. In line with these observations, Singh and co-authors reported abnormal nuclear expression of p53 protein in cervical dysplasia, with a clear association with HPV16/HPV18 infection. The intensity of p53 immunoreactivity varied across different cytological grades of cervical smears. The authors suggested that detection of p53 protein in cervical smears may serve as a diagnostic marker for early-stage HPV-associated cervical cancer [57]. Similarly, Oka and colleagues analyzed the relationship between p53 expression and patient survival before and after irradiation treatment. Their results indicated that high p53 protein levels are significantly associated with poor prognosis [78]. In a subsequent study, the same group demonstrated that the 5-year disease-free survival rate was significantly lower in p53-positive patients compared with p53-negative patients [79].

### 1.2. Low p53—Poor Prognosis

A smaller number of studies suggest that low p53 levels in tumor tissues are associated with poor prognosis. For example, Petrov and colleagues [80] reported that, although higher p53 protein levels were observed in metaplasia, dysplasia, and tumor tissues compared with normal tissue, poorer clinical outcomes were associated with low p53 expression in tumors [80]. Furthermore, it has been suggested that the prognostic impact of p53 expression may depend on tumor stage and degree of differentiation, indicating that the relationship between p53 levels and patient outcome may be context-dependent [57].

### 1.3. No Correlation Between p53 Protein Levels, Tumor Severity, and Patient Survival

A substantial number of studies indicate that p53 protein levels in cervical carcinoma tissues cannot be used as a reliable indicator of patient survival. For example, Wu and colleagues reported no significant correlation between p53 expression and cervical neoplasia grade or patient survival. However, in this study, only approximately 10% of the analyzed lesions (19 out of 203) were classified as invasive cancer, while the majority represented preneoplastic lesions [81]. Similarly, Ngan and colleagues analyzed 107 patients with squamous cell carcinoma of the cervix and found that p53 overexpression in tumor tissues had no prognostic significance [65]. These findings were further supported by Dellas and colleagues, who reported that neither the presence nor the level of p53 protein was associated with overall survival in patients with invasive cervical carcinoma [66]. In another study, Dimitrakakis and colleagues analyzed 171 cervical specimens, including normal epithelium, HPV-associated lesions, cervical intraepithelial neoplasia (CIN), and invasive carcinomas, using immunohistochemical staining. No prognostic significance of p53 expression was identified [75]. Similarly, Hunt and colleagues found in a study with 82 cases of cervical carcinoma no association between p53 expression and tumor subtype, lymph node status, or clinical outcome. The authors concluded that p53 analysis is unlikely to be useful for prognostic assessment in cervical cancer [51]. Comparable results were reported by Haensgen and colleagues, who investigated the relationship between p53 expression, tumor hypoxia, and prognosis. Although hypoxic tumors more frequently exhibited p53 positivity, no correlation was found between p53 levels and patient survival [76]. In addition, Bilgin and colleagues [70] evaluated the expression of p16 and p53 in cervical intraepithelial neoplasia and non-neoplastic cervical lesions through immunohistochemistry. While p16 proved useful in identifying high-grade lesions, p53 expression showed no correlation with lesion severity or patient outcome [70]. Furthermore, Yamashita and colleagues analyzed the expression of six cell cycle–associated proteins (p53, p21/WAF1, MIB-1, EGFR, HER2, and BCL-2) in cervical squamous cell carcinoma. Although HER2 expression showed a weak association with overall survival, no significant clinical correlations were observed for p53, p21/WAF1/CIP1, MIB-1, EGFR, or BCL-2 [82].

Viewed together, these findings demonstrate a lack of consensus regarding the diagnostic and prognostic value of p53 protein expression in cervical cancer. We have previously encountered a similar situation in ovarian cancer, where we demonstrated for an analyzed dataset of more than 400 patients that neither *TP53* mutation status nor p53 protein levels alone were able to provide reliable prognostic information, recognizing the contradictory findings in the past [83]. Therefore, in the present study, we aimed to systematically evaluate the diagnostic relevance of *TP53* mutation status and p53 protein expression in cervical carcinoma using a uniform methodological approach across all analyzed cases. In addition, we incorporated available data on protein levels of key p53 target genes to assess their potential value as prognostic markers in this disease.

## 2. Results

### 2.1. In Cervical Carcinoma, Mutation in TP53 Cannot Be Used as a Prognostic Marker

On one hand, it has long been known that only a minority of cervical carcinoma cases are associated with a mutation in *TP53*, whereas in most cases p53 protein is inactivated by the HPV-derived E6 oncoprotein [59,60]. On the other hand, some studies show that non-HPV16/18 patients had poorer survival than HPV16/18 patients [84].

**Figure 1:**
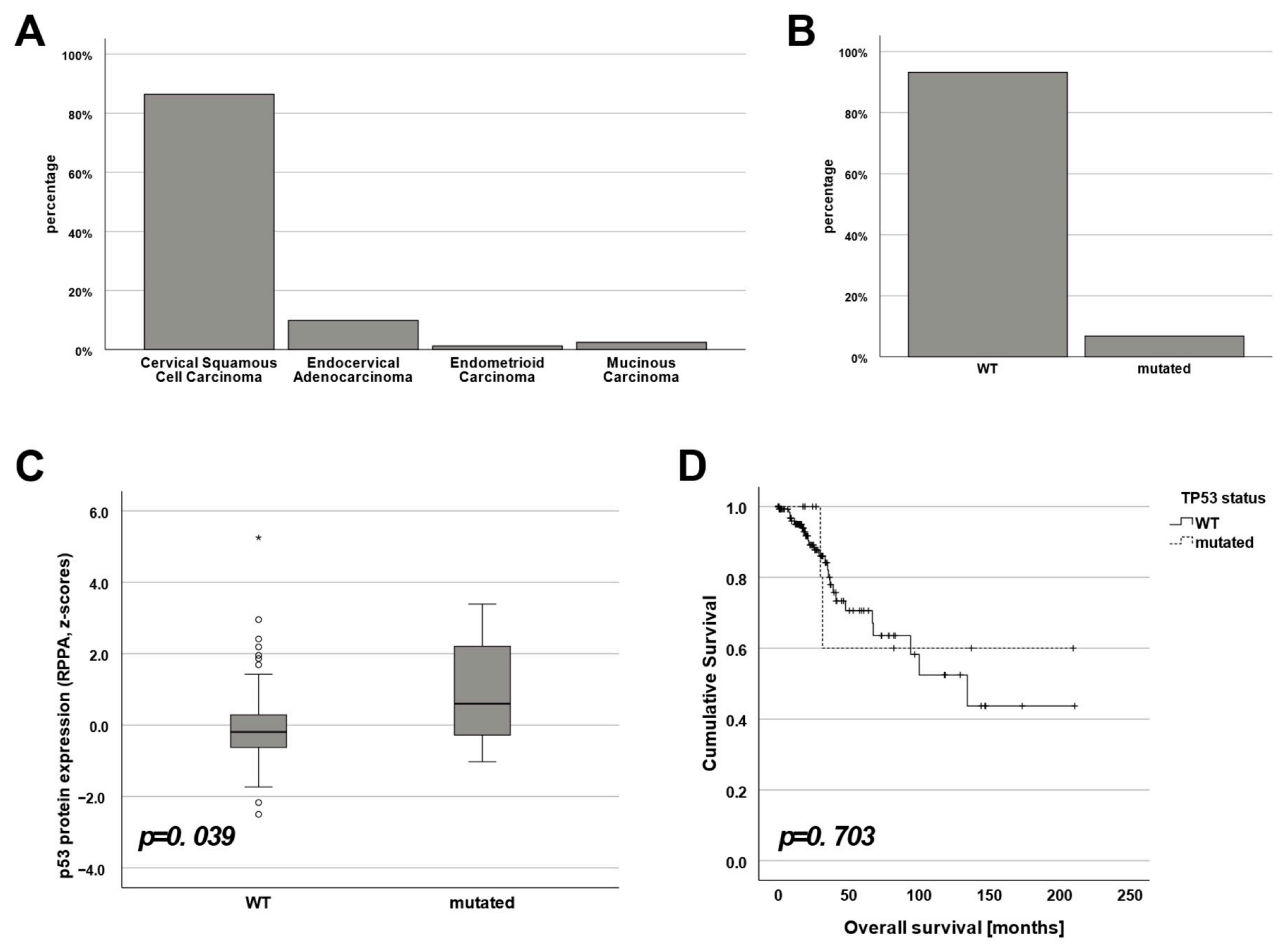
Comparison of p53 protein amount and patients’ survival between cervical tumors without and with mutation in *TP53*. (**A**). Frequency of subtypes of cervical carcinoma in the analyzed cohort. (**B**). Frequency of *TP53* mutation in cervical tumors. (**C**). Comparison of the amount of p53 protein (RPPA, Z-scores; relative values) in cervical tumors without and with mutation in *TP53*. (**D**). Comparison of survival between patients carrying tumors with WT *TP53* and patients carrying tumors with mutated *TP53* (*n* = 151 for WT and *n* = 11 for mutated).

Thus, first we compared the frequency of *TP53* mutation in a cohort of 162 patients suffering from cervical cancer (mainly Cervical Squamous Cell Carcinoma, Figure 1A; more clinical characteristics are displayed in Table 1), and found that, indeed, the majority (151 cases, ~93%) carry WT *TP53* (Figure 1B). This finding is in agreement with the well-established view in the literature indicating that, in contrast to other types of tumors, the main mechanism of p53 inactivation in cervical carcinoma is not mutation but inactivation by the E6 oncoprotein [59,60] as reviewed by Nakamura et al. [85]. In many types of tumors, mutation of *TP53* is accompanied by an increasing amount of p53 protein and a poorer prognosis [86,87,88]. In fact, we have also observed a statistically significant (*U* = 521.00, *Z* = −2.016, *p* = 0.039) increase in the p53 protein amount in the samples from patients suffering from tumors with mutated versions of *TP53* in comparison with tumors without mutation in *TP53* (Figure 1C). Next, we compared the survival rate in the group of patients without *TP53* mutation in tumor tissue with the cohort of patients carrying *TP53* mutant tumors. We have not found any statistically significant correlation (*p* = 0.703, HR = 0.755 (95%-CI 0.177–3.223, *p* = 0.704)) between the *TP53* mutation status and patient survival when comparing the subgroup of mutated p53 with the *TP53*-WT group (Figure 1D). Since only a minority of cases of cervical carcinoma carry mutated *TP53*, we focused the next comparisons on the tumors that carry WT *TP53*.

### 2.2. In Cervical Carcinoma with WT TP53, Determination of p53 Protein Amount Has Only Limited Prognostic Importance

**Figure 2:**
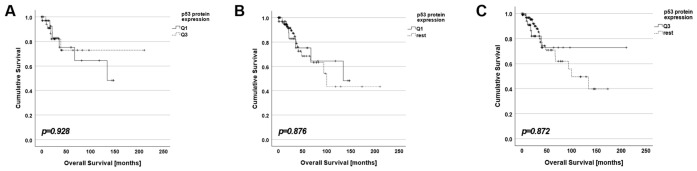
The levels of p53 protein in patients with cervical tumors carrying WT *TP53* are not a valid prognostic marker. (**A**). Comparison of overall survival between patients with the lowest 25% of p53 protein levels in tumors (Q1, *n* = 38) and those with the highest 25% of p53 protein levels (Q3, *n* = 38). (**B**). Comparison of overall survival between patients with the lowest 25% of p53 protein levels in tumors (Q1, *n* = 38) and the rest of the patients (*n* = 113). (**C**). Comparison of overall survival between patients with the highest 25% of p53 protein levels in tumors (Q3, *n* = 38) and the rest of patients (*n* = 113). Group allocations are based on protein levels for the studied cohort of p53-WT patients (*n* = 151).

Since statements regarding the use of the amount of p53 protein as a prognostic marker in cervical carcinoma are contradictory [51,56,58,64,65,66,67,68,69,70,71,72,73,74,75,76,77,78,79,80,81,82,89], we calculated correlations between the amount of p53 protein and survival focusing only on the patients with WT TP53. For this reason, we performed comparisons using the subpopulation of patients who carry WT TP53 as determined by sequencing (Figure 2). As previously performed, we divided the population into subgroups based on their protein levels using the quartiles as cut-off markers [83,90]. By doing so, analyses between the edges themselves as well as comparisons between the top or bottom quartile and the rest of the patients were possible to perform. The total number of analyzed patients was 151.

First, we compared the survival of the 25% of patients with the lowest p53 protein levels in tumors versus the 25% with the highest amount (Figure 2A). In this comparison, no significant difference between the two groups was observed (Log-Rank *p* = 0.928; HR = 1.053, 95%-CI 0.347–3.194, *p* = 0.928).

Next, we compared the cohort of 25% of the patients with the highest p53 protein levels versus the rest of the patients (Figure 2B). In this comparison, we also did not observe any significance when comparing both groups’ survival (Log-Rank *p* = 0.876; HR = 0.931, 95%-CI 0.381–2.280, *p* = 0.876).

Finally, we plotted the data from the 25% of patients with the lowest p53 protein levels versus the rest of the patients (Figure 2C). And similarly to the results presented on panels A and B, we observed no significant difference between the two groups with respect to patients’ survival (Log-Rank *p* = 0.827; HR = 1.079, 95%-CI 0.428–2.716, *p* = 0.872).

In summary: In this type of comparison focusing only on patients carrying WT *TP53*, we again did not observe any statistically significant correlations between the amount of p53 protein and patient survival. This leads us to the conclusion that the determination of p53 protein amount has also only limited diagnostic importance in WT *TP53* cervical carcinomas.

### 2.3. In Cervical Carcinoma with WT TP53, Determination of BAX Protein Amount Seems to Have Prognostic Importance

Since, on the one hand, most patients with cervical carcinoma do not harbor a mutation of *TP53* in tumors, and on the other hand, the BAX gene is a critical transcription target for p53, it was logical to check if the amount of BAX protein is correlated with survival exclusively for the subpopulation of patients with WT *TP53* in tumor tissue.

**Figure 3:**
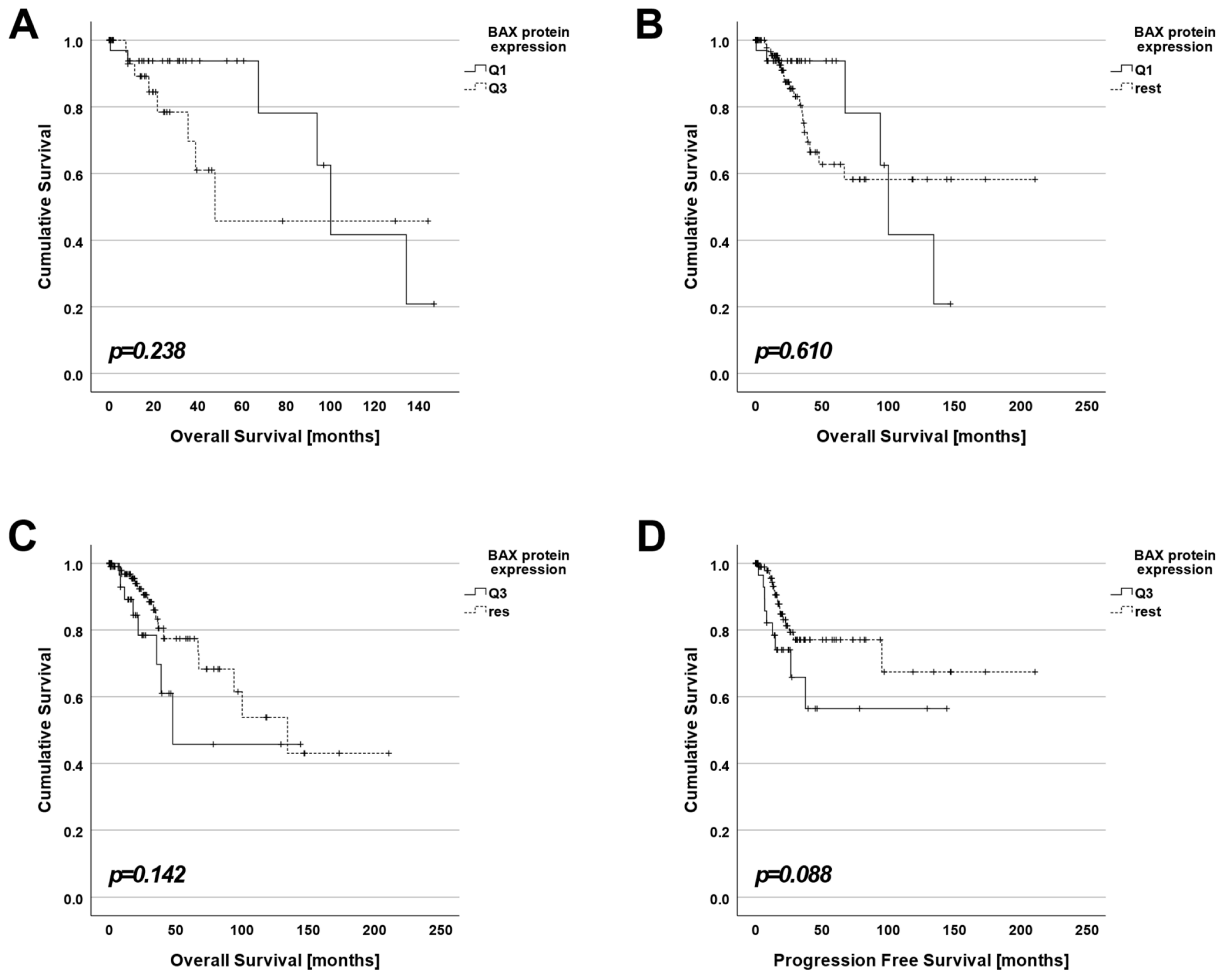
In cervical carcinoma with WT *TP53*, determination of BAX protein amount seems to have prognostic importance. (**A**). Comparison of overall survival between patients with the lowest 25% of BAX protein levels in tumors (Q1, *n* = 38) and those with the highest 25% of BAX protein levels (Q3, *n* = 38). (**B**). Comparison of overall survival between patients with the lowest 25% of BAX protein levels in tumors (Q1, *n* = 38) and the rest of the patients (*n* = 113). (**C**). Comparison of overall survival between patients with the highest 25% of BAX protein levels in tumors (Q3, *n* = 38) and the rest of the patients (*n* = 113). (**D**). Comparison of progression-free survival (PFS) between patients with the highest 25% of BAX protein levels in tumors (Q3, *n* = 38) and the rest of the patients (*n* = 113). Group allocations are based on protein levels for the studied cohort of p53-WT patients (*n* = 151).

We compared whether the protein level of BAX in tumors can be correlated with survival for patients with WT *TP53* (Figure 3). Based on the availability of data, we were able to include datasets of 151 patients for this study. We performed three kinds of analyses by dividing the collective into groups according to the BAX protein level:

Initially, survival was compared between patients whose tumors exhibited the highest quartile of BAX protein expression and those whose tumors exhibited the lowest quartile of BAX expression (Figure 3A). Although a slight tendency towards a negative correlation between BAX protein levels and patients’ survival was observed, the differences did not reach the level of significance (Log-Rank *p* = 0.238; HR = 0.530, 95%-CI 0.182–1.547, *p* = 0.246).

We next compared the survival of patients whose tumors exhibited BAX protein levels within the lowest quartile with that of all remaining patients (Figure 3B). No statistically significant difference in survival was observed between the two groups (Log-Rank *p* = 0.610, HR = 0.787, 95%-CI 0.314–1.977, *p* = 0.611).

As a next step, we compared the survival of patients whose tumors exhibited BAX protein levels within the highest quartile with that of all remaining patients (Figure 3C). This time, a strong tendency towards a difference in survival between the patients in both groups (Log-Rank *p* = 0.142; HR = 1.864, 95%-CI 0.802–4.332, *p* = 0.148) was found, indicating that patients with a high amount of BAX protein have a worse prognosis than patients with lower levels. However, we did not reach the level of significance.

As we have seen signs for a negative correlation between the BAX protein level and patients’ survival, we decided to have a look at another very important parameter, the progression-free survival (PFS). Again, we compared a group of patients whose tumors exhibited BAX protein levels within the highest quartile with that of all remaining patients (Figure 3D). Here, we found an even stronger tendency indicating the disadvantage of high BAX levels. However, with Log-Rank *p* = 0.088, we just missed the level of significance (HR = 1.993, 95%-CI 0.888–4.475, *p* = 0.095).

In summary, in this type of comparison focusing only on patients carrying WT *TP53*, we observed a strong tendency towards a correlation between BAX protein levels and patient survival. Specifically, high BAX protein levels seem to be associated with worse patient survival.

### 2.4. In Cervical Carcinoma with WT TP53, Determination of p21(CDKN1A) Protein Amount Has No Prognostic Importance

**Figure 4:**
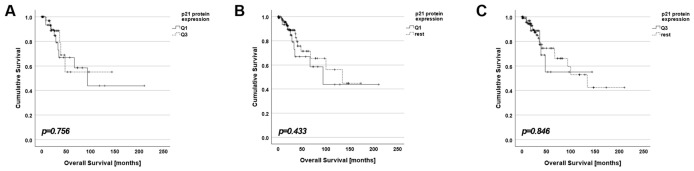
The level of p21 protein in patients with cervical tumors carrying WT *TP53* cannot be used as a valid prognostic marker. (**A**). Comparison of overall survival between patients with the lowest 25% of p21 protein levels in tumors (Q1, *n* = 38) and those with the highest 25% of p21 protein levels (Q3, *n* = 38). (**B**). Comparison of overall survival between patients with the lowest 25% of p21 protein levels in tumors (Q1, *n* = 38) and the rest of the patients (*n* = 113). (**C**). Comparison of overall survival between patients with the highest 25% of p21 protein levels in tumors (Q3, *n* = 38) and the rest of the patients (*n* = 113). Group allocations are based on protein levels for the studied cohort of p53-WT patients (*n* = 151).

Since p21 is one of the best described transcription targets for p53, we calculated correlations between the amount of p21 protein and survival, focusing only on the patients with WT *TP53*. For this reason, we performed comparisons using the subpopulation of patients who carry WT *TP53* as determined by sequencing (Figure 4). The total number of analyzed patients was 151, divided into three groups:

First, we compared the survival of patients with the lowest 25% of p53 protein levels in tumors versus those with the 25% highest amounts (Figure 4A). In this comparison, we have not observed any tendency towards a correlation between the amount of p21 protein and the survival of patients (Log-Rank *p* = 0.756; HR = 1.181, 95%-CI 0.415–3.361, *p* = 0.756).

Next, we plotted the data from patients with the lowest 25% of p53 protein levels versus the rest of the patients (Figure 4B). And similarly to the results presented in panel A, we have not observed any statistically significant difference between both groups (Log-Rank *p* = 0.433; HR = 1.387, 95%-CI 0.610–3.152, *p* = 0.435).

Finally, we compared the cohort of patients with the highest 25% of p21 protein levels versus the rest of the patients (Figure 4C). Also in this comparison, we did not observe any statistically significant difference in survival (Log-Rank *p* = 0.846; HR = 1.096, 95%-CI 0.434–2.769, *p* = 0.846).

In summary: In this type of comparison focusing only on patients carrying WT *TP53*, we have not observed any statistically significant correlations between the amount of p21 protein and patient survival. This leads us to the conclusion that the determination of p21 protein amount has no diagnostic importance in WT *TP53* cervical carcinomas by now.

### 2.5. In Cervical Carcinoma, Amount of Total TIGAR Protein Is Not Significantly Associated with Patients’ Prognosis

**Figure 5:**
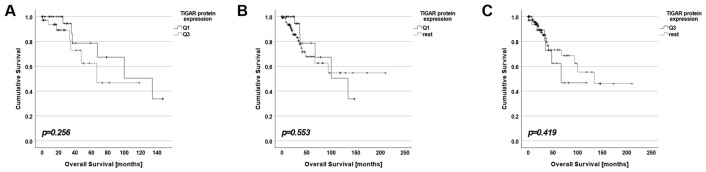
In cervical carcinoma, total TIGAR protein levels show no significant correlation with patients’ survival. (**A**). Comparison of overall survival between patients with the lowest 25% of TIGAR protein levels in tumors (Q1, *n* = 38) and those with the highest 25% of TIGAR protein levels (Q3, *n* = 38). (**B**). Comparison of overall survival between patients with the lowest 25% of TIGAR protein levels in tumors (Q1, *n* = 38) and the rest of the patients (*n* = 113). (**C**). Comparison of overall survival between patients with the highest 25% of TIGAR protein levels in tumors (Q3, *n* = 38) and the rest of the patients (*n* = 113). Group allocations are based on protein levels for the studied cohort of p53-WT patients (*n* = 151).

To investigate the potential prognostic relevance of TIGAR expression, we analyzed the relationship between tumor TIGAR protein levels and overall patient survival (Figure 5). Data from 151 patients were available for this analysis. Patients were stratified according to TIGAR protein abundance, and survival outcomes were evaluated using three complementary approaches.

First, overall survival was compared between patients with tumors exhibiting TIGAR levels within the upper quartile and those within the lower quartile (Figure 5A).

Next, the subgroup of patients characterized by the lowest quartile of TIGAR expression was compared with all remaining patients in the cohort (Figure 5B).

Finally, survival was assessed in patients whose tumors belonged to the highest quartile of TIGAR expression and compared with the rest of the study population (Figure 5C).

While there was no significant difference (or even tendency) at all in the overall survival when comparing patients that have tumors with the lowest 25% (Log-Rank *p* = 0.553, HR = 0.757, 95%-CI 0.300–1.909, *p* = 0.555) or the highest 25% (Log-Rank *p* = 0.419; HR = 1.439, 95%-CI 0.592–3.493, *p* = 0.422) of p53 protein levels versus the rest of patients we found at least a slight tendency in difference in the survival rate when comparing the subgroup of patients that have tumors with the lowest 25% and the group with the highest 25% of TIGAR protein levels. In this analysis, the Q1-group showed enhanced survival compared to the Q3-group (Log-Rank *p* = 0.256; HR = 0.513, 95%-CI 0.159–1.654, *p* = 0.264). Since none of the *p*-values reached statistical significance, we can only conclude that high total TIGAR protein levels may represent a moderate indicator of poor prognosis in cervical carcinoma.

## 3. Discussion

The protein encoded by the *TP53* gene—the tumor suppressor p53—is one of the most important and extensively studied factors in both tumorigenesis and anticancer treatment. According to the National Library of Medicine (NLM) database, the world’s leading biological and medical library, more than 130,000 related records have been identified, and this number continues to grow. Other genes or proteins that are also considered important in tumor biology have substantially fewer associated records. The p53 protein plays a central role in regulating cellular responses to various stimuli and stress signals [4,5,6,7]. This has naturally led to efforts to establish correlations between various p53-regulated parameters and patient survival. As mentioned in the Introduction, numerous researchers and clinicians have attempted to use *TP53* mutation status and p53 protein levels as prognostic markers in cervical cancer. However, the reported findings remain highly inconsistent [51,56,58,61,62,64,65,66,67,68,69,70,71,72,73,74,75,76,77,78,79,80,81,82,89].

The main reasons for such variability include differences in experimental approaches and data analysis methods. To address this issue, we used data from The Cancer Genome Atlas (TCGA) research network, which provides extensive protein datasets generated using reverse-phase protein array (RPPA). The strength of this method lies in its underlying principle. RPPA is a high-throughput, antibody-based targeted proteomics platform that enables the quantification of hundreds of proteins across thousands of samples derived from cell lysates, tissues, or fluids. Protein samples are robotically arrayed onto nitrocellulose-coated glass slides, and each slide is probed with a specific antibody capable of detecting various parameters, including total protein expression levels and post-translational modifications [91]. Afterwards, the obtained data are normalized (Z-Scores). This approach allows for more precise quantification of protein abundance. For our analysis, we utilized the cBioPortal database (https://www.cbioportal.org; accessed on 8 July 2026) [89,92,93], which offers a comprehensive collection of cancer-related datasets. Previous studies have shown that only a small percentage of cervical carcinoma patients harbor mutations in the *TP53* gene, whereas in most cases, p53 protein is inactivated by the HPV-derived E6 oncoprotein [59,60]. Moreover, it has been reported that patients with non-HPV16/18 tumors have poorer survival compared with those with HPV16/18-associated tumors [84]. The association and clinical significance of *TP53* gene status and p53 protein levels in gynecological cancers have been comprehensively reviewed by Nakamura et al. [85]. In agreement with these studies, we found that the majority of cervical carcinoma patients (~93%) harbor tumors with wild-type *TP53*. Moreover, our results indicate that *TP53* mutation status cannot be used as a prognostic factor in cervical carcinoma. Previous studies have shown that, in many types of human cancer, *TP53* mutations are associated with increased p53 protein levels and poorer patient prognosis [86,87,88], which was also the case for the analyzed data set in our case. Since the majority of cervical carcinoma cases harbor wild-type *TP53*, we next focused on evaluating the relationship between p53 protein levels and patient survival within this subgroup. As noted above, this issue remains highly controversial [51,56,58,61,62,64,65,66,67,68,69,70,71,72,73,74,75,76,77,78,79,80,81,82,89]. We did not find any statistically significant correlations between p53 protein levels and patient survival. In conclusion, our findings indicate that the determination of p53 protein levels has only limited diagnostic value in cervical carcinomas harboring wild-type *TP53*. That said, we want to highlight that our finding on the limited prognostic relevance of *TP53*-status does not interfere with the molecular importance of a *TP53* mutation in cancer development as described carefully in the introduction but adds a new perspective on the clinical relevance in respect to patients’ prognosis. Apoptosis is regulated by a balanced interaction between various pro- and anti-apoptotic proteins, among which BCL-2 and BAX are key regulators [94,95]. In many tumor types, it is well established that the balance between BCL-2 and BAX expression determines the sensitivity of tumor cells to apoptosis [96,97,98,99,100]. In particular, BAX overexpression has been shown to enhance susceptibility to apoptosis, whereas elevated levels of BCL-2 are associated with tumor development and progression [100]. In a study conducted by Oliveira and colleagues, both cervical microinvasive carcinoma and CIN III samples exhibited stronger and more widespread BAX expression compared with BCL-2 staining, suggesting a shift toward pro-apoptotic signaling [63]. However, previous studies have shown that BAX protein immunostaining does not differ significantly between CIN III lesions and cervical cancer samples [101,102,103], indicating that BAX expression does not consistently correlate with the induction of apoptosis in cervical carcinoma [103]. In general, the role of BAX protein levels in cervical carcinoma and their potential use as a prognostic marker in cancer treatment remain controversial [63,104,105,106,107,108,109,110,111,112,113,114,115,116,117]. Therefore, we performed our own analysis using the cBioPortal database (https://www.cbioportal.org; accessed on 8 July 2026) [89,92,93]. Our study demonstrates that, in cervical tumors harboring wild-type *TP53*, there is a strong tendency towards an inverse correlation between BAX protein levels and patient survival. Specifically, elevated BAX expression seems to be associated with poorer prognosis. At first glance, our finding that elevated BAX protein levels are associated with poorer patient survival may appear counterintuitive, considering the well-established pro-apoptotic function of BAX. However, the prognostic significance of BAX expression remains controversial. Conflicting results have been reported not only across different tumor types but, in some cases, even within the same malignancy. For example, Wang et al. [118] demonstrated that elevated BAX expression is associated with unfavorable prognosis across multiple human cancers. In contrast, reduced BAX expression has been associated with poor clinical outcome in epithelial ovarian cancer [119] and esophageal squamous cell carcinoma [120,121]. Moreover, even within the same tumor type, conflicting findings regarding the prognostic significance of BAX have been reported. For example, studies on esophageal squamous cell carcinoma have reached inconsistent conclusions concerning the association between BAX expression and patient outcome [120,122,123,124]. This heterogeneity is consistent with subsequent systematic reviews demonstrating substantial inter-study variability in prognostic gene expression markers in esophageal cancer [125]. Taken together, these findings indicate that the prognostic significance of BAX cannot be generalized and should be interpreted in a tumor-specific context. In this regard, our study provides additional evidence that, in cervical carcinoma harboring wild-type *TP53*, elevated BAX protein expression is associated with poorer patient survival. Given the conflicting findings previously reported for cervical cancer, our analysis of the TCGA RPPA dataset strengthens the evidence supporting BAX as a potential prognostic biomarker in this disease. Nevertheless, the molecular mechanisms underlying this association remain to be elucidated in future functional studies. These findings contribute to a more comprehensive understanding of cervical cancer pathogenesis and may facilitate more accurate prognostic assessment, as well as inform the development of improved therapeutic strategies. In contrast to the clearly demonstrated diagnostic value of BAX protein, our results indicate that the levels of both p21 and TIGAR have little to no diagnostic significance. In the case of p21, our findings are consistent with those reported by Yamashita and colleagues [82], who demonstrated that the expression level of p21/WAF1/CIP1 is not associated with prognostic value or treatment planning. Similar conclusions regarding the limited prognostic relevance of p21/WAF1/CIP1 expression have been reported by other groups [79]. In contrast, detailed studies addressing the diagnostic role of TIGAR in cervical cancer remain limited.

Although we are convinced that our study provides substantial new insights into the prognostic relevance of p53 and p53-target genes, we acknowledge our study’s limitations. First of all, as shown above, the fraction of *TP53*-mutated cancer patients is, in the case of cervical cancer, very low. Consequently, due to limited statistical power, almost no robust conclusions can be made for this subgroup. Furthermore, for the analyzed data set we were not able to obtain any information on the HPV status. As HPV infection is one of the main mechanisms in the development of cervical cancer, an analysis comparing HPV+ and HPV− patients would be very interesting. Due to the exploratory approach focusing on the statistical analysis of the prognostic impact of certain proteins, it is not possible to directly draw conclusions on the underlying molecular mechanisms or the molecular importance of the respective proteins. With respect to our results regarding the importance of BAX as a prognostic marker, a repetition of this analysis once more data are available seems to be worthwhile in order to verify the observed tendency. Moreover, it seems worthwhile to divide the population of cervical cancer patients into more subgroups for further analysis based on information such as cancer stage, pretreatment, patient age, etc., as soon as enough data for a robust statistical analysis are available.

## 4. Materials and Methods

**Figure 6:**
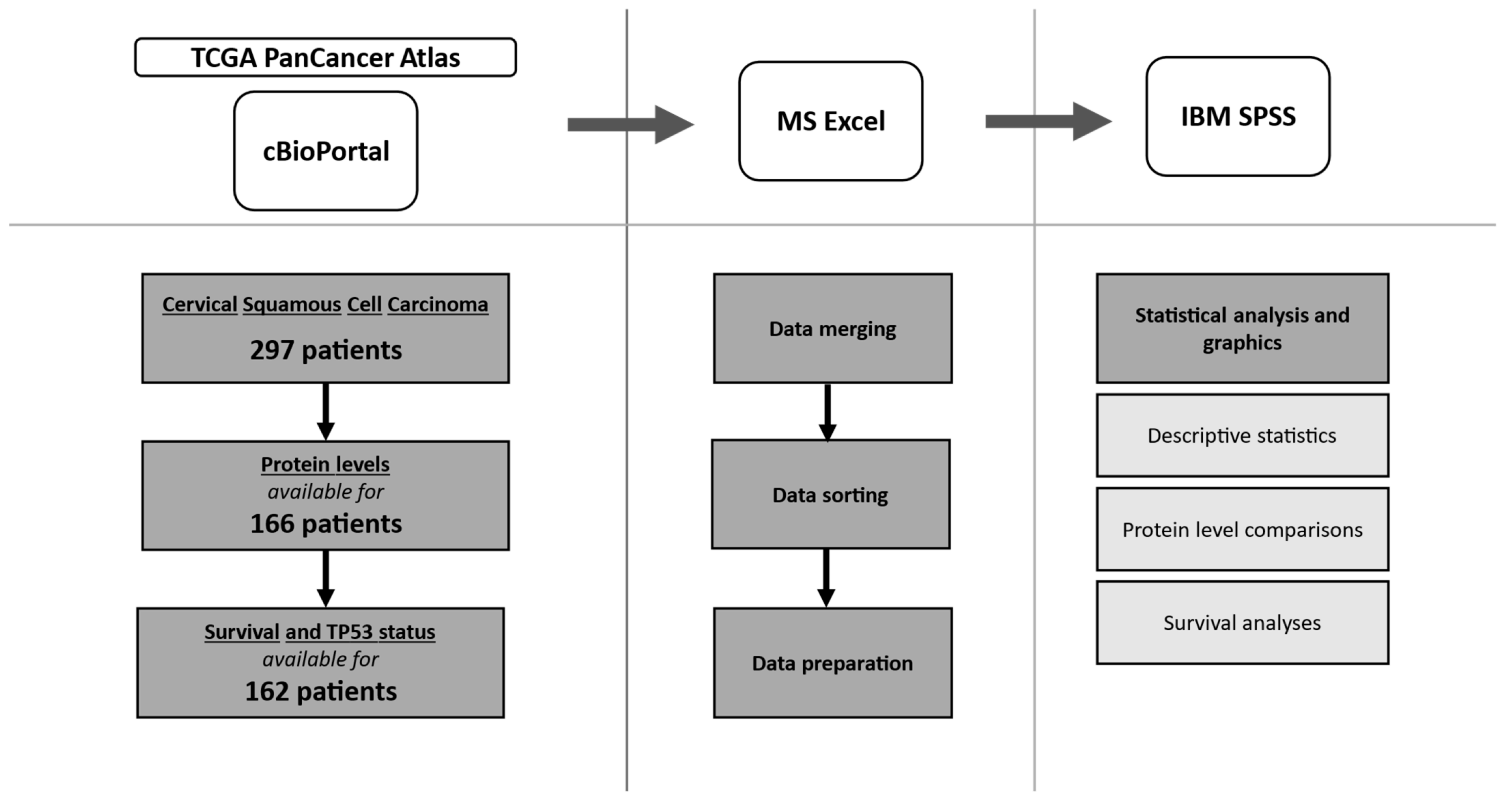
Workflow for data extraction and statistical analysis. Data from the TCGA PanCancer Atlas were obtained via cBioPortal v7.0.5 and selected for the respective criteria. Further data processing was performed in MS Excel before carrying out statistical analyses in IBM SPSS Statistics Version 31.0.0.0.

We extracted data from The Cancer Genome Atlas (TCGA) PanCancer Atlas database (https://gdc.cancer.gov/about-data/publications/pancanatlas, accessed on 8 July 2026; hosted by the National Institutes of Health (NIH), Bethesda, MD, USA), finally accessed via cBioPortal v7.0.5 (https://www.cbioportal.org; hosted by the Computational Biology Center, Memorial Sloan-Kettering Cancer Center, New York, NY, USA; accessed on 8 July 2026) [89,92,93] in the same manner as previously performed and published for ovarian cancer [83]. We selected a collective of 162 patients based on the availability of information regarding both protein levels for p53,the three prominent p53 target genes and the *TP53* mutation status, as well as information on the overall survival (OS) and the progression-free survival (PFS); Date of accession: 8 July 2026. After data extraction, the datasets were merged and consolidated in Microsoft Office Excel (Microsoft Corporation, Redmond, WA, USA) before transfer to IBM SPSS Statistics Version 31.0.0.0 (IBM Corporation, Armonk, NY, USA) for statistical analysis. Protein values for p53 were compared for all 162 patients using the Independent Samples Mann–Whitney U test. We preferred this test over the *t*-test because the case groups had a large difference in sample size and did not exhibit complete normal distribution. For the survival analyses based on p53 and p53 target gene protein levels, we divided the collective of patients with WT *TP53* tumors (*n* = 151) into groups in accordance with the quartiles for the p53 protein level (RPPA, Z-scores) of the selected cohort [126]. Survival analyses were displayed by Kaplan–Meier plots using the Log-Rank test to check for statistical significance. Hazard Ratios (HR) were calculated by using p53-WT in Figure 1, Q3 in Panel (A) in Figure 2, Figure 3, Figure 4 and Figure 5 and the subgroup “rest” in all other survival analyses as reference category. The precise number of included patients per group is mentioned in the respective result section. As an indication for statistical significance, we used a *p*-value lower than 0.05 in our comparisons. A summary of the workflow can be found in Figure 6.

## 5. Conclusions

The study of p53 in anticancer therapy remains one of the most important areas in cancer research. However, in cervical carcinoma, both *TP53* mutation status and p53 protein levels appear to have limited value as prognostic markers. Similarly, the p53-regulated proteins p21 and TIGAR show minimal prognostic relevance.

In contrast, downstream effectors of p53 signaling—particularly BAX—may provide meaningful prognostic insights in tumors retaining wild-type *TP53*.

## Figures and Tables

**Table 1:** Clinical characteristics of the analyzed collective of patients.

		Total		*TP53* WT Only		*TP53* Mutated Only	
		Abs.	%	Abs.	%	Abs.	%
**Cases analyzed**		162	100	151	93.2	11	6.8
**Mean Age at diagnosis [Years]**		47.35		46.38		59.09	
**Tumor Stage (UICC)**	NA	83	51.2	78	51.7	5	45.5
	I	44	27.2	41	27.2	3	27.3
	II	13	8.0	12	7.9	1	9.1
	III	18	11.1	16	10.6	2	18.2
	IV	4	2.5	4	2.6	0	0
**Histologic Grade**	NA	5	3.1	5	3.3	0	0
	GX	8	4.9	8	5.3	0	0
	G1	9	5.6	9	6.0	0	0
	G2	69	42.6	66	43.7	3	27.3
	G3	70	43.2	62	31.1	8	72.7
	G4	1	0.6	1	0.7	0	0

## Data Availability

The results shown here are, on the whole, based on data generated by the TCGA Research Network: https://www.cancer.gov/tcga; accessed on 8 July 2026. More information on how the data were accessed can be found in the Materials and Methods Section.
